# Mothers experience on neonatal danger signs and associated factors in northwest Ethiopia: a community based cross-sectional study

**DOI:** 10.11604/pamj.2022.41.83.32176

**Published:** 2022-01-31

**Authors:** Zemene Tigabu Kebede, Alemayehu Teklu Toni, Ashenafi Tazebew Amare, Tadesse Awoke Ayele, Tesfahun Melese Yilma, Tadesse Guadu Delele, Gashaw Andargie Biks, Kassahun Alemu Gelaye

**Affiliations:** 1 Departments of Pediatrics and Child Health, School of Medicine, College of Medicine and Health Sciences, University of Gondar, Gondar, Ethiopia,; 2 Departments of Epidemiology and Biostatistics, Institute of Public Health, College of Medicine and Health Sciences, University of Gondar, Gondar, Ethiopia,; 3 Department of Health Informatics, Institute of Public Health, College of Medicine and Health Sciences, University of Gondar, Gondar, Ethiopia,; 4 Department of Environmental and Occupational Health, and Safety, Institute of Public Health, College of Medicine and Health Sciences, University of Gondar, Gondar, Ethiopia,; 5 Departments of Health System and Policy, Institute of Public Health, College of Medicine and Health Sciences, University of Gondar, Gondar, Ethiopia

**Keywords:** Experience, knowledge, neonate, danger sign, Ethiopia

## Abstract

**Introduction:**

even though there is a significant decline in neonatal mortality globally, it remained unacceptably high in Ethiopia. The estimated experience of neonatal danger signs affects the outcome more than the perceived knowledge. The main aim of this study was to estimate the experience of mothers on neonatal danger signs and its associated factors in Northwest Ethiopia.

**Methods:**

a community-based cross-sectional study was conducted from April 6-16, 2019. All the women who have delivered live birth in the past six months in three districts of Northwest Ethiopia were the source populations. A total of 2424 mothers were selected using two-stage stratified cluster random sampling technique. A pretested and semi-structured interviewer-administered questionnaire was used to collect data from eligible mothers. A multivariable logistic regression model was used to identify independent factors that affected mother´s experiences about neonatal danger signs at a p-value of 5%.

**Results:**

in this study, 2335 (96.3%) mothers completed the interview and 1509 (64.6%) of them have mentioned at least one danger sign. However, only 160 (11.0%) mothers have experienced danger signs in their babies. Of these, about 54 (49.1%) mothers have noticed within 24 hours of delivery and 37 (33.6%) have noticed after 48 hours of delivery. Fifty (45.5%) mothers have noticed the danger signs at home after birth, and 48 (43.6%) have noticed during birth. The frequently reported danger signs were; baby feels hot 106 (66.3%), fast breathing 67(41.9%), and difficulty of breathing 61(38.1%). Mothers who are living in urban, AOR=1.8(95%CI: 1.04,3.0), having multiple pregnancy, AOR=9.8 (95%CI: 2.3,42.0), absence of obstetric danger signs or complication, AOR=0.4 (95%CI: 0.2,0.6), post-term gestational age, AOR=6.5 (95%CI: 2.1,19.5), preterm gestational age, AOR=3.3 (95%CI: 0.8,13.4), assessment by hospital staff during delivery, AOR=2.1 (95% CI: 1.01,4.3), and poor mothers knowledge on neonatal danger signs, AOR=0.7 (95% CI: 0.5,0.9) were the predictors of mothers experience on neonatal danger signs.

**Conclusion:**

even though the knowledge of mothers on neonatal danger signs is high, the practice or experience in using their knowledge is very low. We recommend an implementation study to be conducted to bridge this “know-do” gap.

## Introduction

The neonatal period (birth to one month) is the most critical period for child survival. Globally, nearly 2.5 million neonates died in 2017. Seventy-nine percent of these deaths occurred in countries like Sub-Saharan Africa and South Asia, where there are poor resource settings [1, 2]. Even though there is a significant decline globally, neonatal mortality has remained unacceptably high in Ethiopia for the past two decades [3]. The primary causes of these deaths are due to preterm birth, intrapartum-related complications, infections and birth defects [4]. Global strategies to reduce neonatal mortality include identification of neonatal danger signs and early seeking of prompt medical care if necessary. For effective identification of neonates at risk of morbidity and mortality, and seek appropriate level of healthcare, mother´s recognition of danger signs is necessary [4-6]. UNICEF and WHO define the following signs and symptoms as danger signs in newborns: (1) feeding problem (2) convulsions, (3) fast breathing, (4) severe chest in-drawing, (5) fever, (6) feeling cold (7) movement only when stimulated, or not even when stimulated, (8) yellow soles, (9) reddened or pus draining umbilicus and, (10) reddened or pus draining eyes. These symptoms are believed to be easily recognizable by community health workers or possibly mothers [7].

The knowledge of mothers of neonatal danger signs varies globally, continentally and nationally. In Saudi Arabia, 37% of mothers knew three or more danger signs [8]. In rural Uganda, only 14.8% of mothers identified more than 2 danger signs [9]. Mothers knowledge on neonatal danger signs varies greatly among study findings in Ethiopia; 20.3% in Ambo [10], 18.2% in Gondar town [11], 21.7% in Woldia Hospital [12], 31.3% in Wolkite town [13], 43.7% in Aksum town [14], 50.3% in Chencha district [15], 50.6% in Mekelle city [16]. And it is 26.3% in Nepal [17] and 15.5% in Kenya [18]. Scientific evidences indicated that different factors were affecting the mother´s knowledge on neonatal danger signs. Mothers´ and husbands´ higher educational achievement, living close to health facilities, previous experience of neonatal danger signs, ANC and PNC attendance and access to television for information were positively associated with mothers´ good knowledge about neonatal danger signs [5, 9-13]. The absence of specific clinical manifestations of various neonatal illnesses were resulting in high neonatal morbidity and mortality in Ethiopia and other low income countries [13-16, 19]. A study done among mothers from Ghana who had given birth within the preceding two years to identify factors associated with neonatal danger signs. This study found that maternal delivery complications, neonatal complications within the first six weeks of life, maternal age less than 20 years, maternal education level lower than secondary school, and fewer than four antenatal care visits have significantly predicted neonatal danger signs [19]. A study from Ethiopia pointed out that low birth weight and maternal danger signs during pregnancy and delivery is associated with neonatal danger signs [20].

Another study done in Egypt among high risk mothers during perinatal period has found relationship between neonatal danger signs and maternal characteristics, such as mother´s educational level, maternal age, occupation, parity, and maternal antenatal danger signs [21]. This study conducted among high risk mothers and failed to identify the strength of association between neonatal danger signs and maternal characteristics. Although there are efforts to identify factors associated with neonatal danger signs, there are variations in the study setting, time, and results. The study in Ghana was conducted among mothers from Ghana who had given birth within the preceding two years in 2013 which might have recall bias. The study in Ethiopia identified only birth weight and maternal complications as factors associated with neonatal danger sign. The study in Egypt identified correlations of danger signs with maternal characteristics but failed to determine strength of association. In our study, we identify the associated factors of neonatal danger as well as determined the strength of association. The experience of mothers can positively influence health-seeking intention, which has a major impact on the reduction of neonatal mortality. Household-level women empowerment, perception toward the behavior of healthcare providers, and the perceived cost of treatment had a positive association with treatment-seeking intention [20]. The potential determinants of the experience of mothers on neonatal danger signs and factors that would delay for sick newborn treatment were categorized into four domains: maternal factors, family factors, antenatal factors, and delivery factors [19]. However, few studies conducted previously regarding mothers´ experience on neonatal danger signs reported that 24.9% in northwest Ethiopia [20], 65.1% in Wolkite town [13], and 60.5% in Ambo town [10] from Ethiopia. These findings revealed the inconsistency across studies regarding mothers´ real experience on neonatal danger signs. In Ethiopia, like Uganda, Ghana, Nigeria and Saudi Arabia, significant works has been done regarding mothers´ level of knowledge of neonatal danger signs and their determinant factors [5, 6, 8-12, 19, 20, 22]. Nonetheless, evidence on the mothers´ real experience on their neonates is scarce. Therefore, the main aim of this study was to estimate the experience of mothers on neonatal danger signs and associated factors in Northwest Ethiopia.

## Methods

### Study design and area

A community-based cross-sectional study was conducted in three randomly selected districts of North and Central Gondar Zones of Ethiopia from April 6-16, 2019. The study districts were Dabat, Debark, and Wogera which are located in the northwest part of Ethiopia. According to the Central Statistical Agency (CSA), in 2017, the total projected population was 3,654,920 of which 1,847,631 (51%) were males [23]. These zones have three government hospitals, 133 health centers and 563 health posts that provide primary healthcare for the population. Health post is the smallest primary healthcare unit in the three-tier healthcare system of Ethiopia.

**Source and study population:**all women who had delivered live birth in the past six months were the source populations. The study participant women were randomly selected from clusters in Dabat, Debark and Wogera districts.

### Sample size and sampling strategy

The total sample of the study was determined using statcalc in EpiInfo version 7.1.5.0 with assumptions of 3% level of significance, 3% margin of error and taking 32.9% proportion of good maternal knowledge on neonatal danger signs [24]. By considering a 5% non-response rate and design effects of two, a total of 2424 women were included in the study. A multistage stratified cluster sampling technique was used to select the mothers. First, clusters were randomly selected from three districts (Debark, Dabat, and Wogera) proportional to the size of clustersin the districts. Then, villages were randomly selected from the selected clusters.

### Variables and operational definitions

Knowledge and experience of mothers on neonatal danger signs and treatment outcome were the outcome variables. Socio-demographic factors including age, sex, residence, religion, educational status, marital status, occupation, income level and obstetric and delivery characteristics including birth weight, parity, ANC attendance, pregnancy interval, birth history, gestational age, delivery place, and delivery outcome were considered as predictors. Knowledge of mothers on neonatal danger signs was defined as symptoms that complicate the lives of the neonate and happen during the neonatal period. Based on knowledge of neonatal danger signs, mothers who were able to mention at least one sign were considered as “Good” whereas those who were unable to mention at least one were considered as “Poor”. The experience of mothers on neonatal danger signs was defined as it was an event on mothers which leaves an impression about the neonate during the last six months´ time. Based on experience, mothers who had experienced at least one neonatal danger sign were considered as ‘Yes’ whereas those who had no experience were considered as ‘No’.

### Data collection procedures

The data were collected using a pretested questionnaire designed using a tablet-based data collection application called Open Data Kit (ODK) software. A semi-structured interviewer-administered questionnaire adopted from previous literature [8-11, 19, 20, 24] were used to record the mother´s knowledge and experience about neonatal danger signs, and other related factors. The questionnaire was translated into the local language (Amharic) and translated back to the English version to check the content validity of the original version. Two days of training had been given for data collectors and supervisors before data collection. Regarding the training, the emphasis was given on purposes of the study, appropriate meanings of each question and ways of communication. To reduce a recall bias, the interviewers mentioned each danger signs one by one and gave mothers adequate time to respond properly.

### Data processing and analysis

All the electronic data were downloaded and transferred to STATA version 14 software for analysis. Data quality was checked by running frequencies, proportions, and summary statistics. Descriptive statistics were used to describe the study population with relevant variables and presented using tables. A multivariable logistic regression model was used to identify independent factors that affected the mothers´ experiences on neonatal danger signs at a p-value of 5%.

## Results

### Socio-demographic characteristics of respondents

From the total of 2424 sampled mothers, 2335 have completed the interview, forming a response rate of 96.3%. The majorities of the mothers were aged 25-29, 655(28.1%), and followed by 20-24, 547(23.4%) years old. Among the mothers, about 1687(72.3%) were from rural areas, and 2219(95.0%) were married. Only 835(35.8%) mothers attended formal education. Of these, the majorities of 576 (69%) mothers have attended only primary level education. Of the total, 1304 (55.8%) mothers were living within five kilometers radius from the nearest health center (Table 1).

**Table 1:** socio-demographic characteristics of respondents, April 2019, northwest Ethiopia (p-value=0.05)

Variables	Number (%)	95% CI (%)
**Maternal age (years)**		
15-19	149(6.4)	5.5,7.5
20-24	547(23.4)	21.8,25.2
25-29	655(28.1)	26.3,29.9
30-34	468(20.0)	18.5,21.7
35-39	381(16.3)	14.9,17.9
40-44	106(4.5)	3.8,5.5
45-50	29(1.2)	0.9,1.8
**Place of residency**		
Urban	313 (13.4)	12.1,14.9
Semi-urban	335 (14.4)	12.9,15.8
Rural	1687 (72.3)	70.4,74.0
**Marital status**		
Single	67 (2.9)	2.3,3.6
Married	2219 (95.0)	94.1,95.8
Others	49 (2.1)	1.6, 2.8
**Mother´s occupation**		
Housewife	1029 (44.1)	42.1,46.1
Farmer	1146 (49.1)	47.05,51.1
Merchant	50 (2.1)	1.6,2.8
Others	110 (4.7)	3.92,5.65
**Mothers education attendance**		
No	1500 (64.2)	62.3,66.2
Yes	835 (35.8)	33.8, 37.7
Educational status of mother (n=835)		
Primary education	576 (69)	65.8, 72.0
Second education	232 (27.8)	24.8,30.9
College and above	27 (3.2)	2.2,4.7
**Distance to the nearest health center**		
≤1 kilometer	571 (24.5)	22.8,26.2
1.1-5 kilometer	733 (31.4)	29.5,33.3
5.1-10 kilometer	578 (24.8)	23.0,26.6
11.0-20 kilometer	453 (19.4)	17.9,21.1
**Time to reach to the nearest health center**		
≤ 60 minutes	1526 (65.4)	63.4,67.3
61-180 minutes	787 (33.74)	31.8,35.7
≥ 181 minutes	22 (0.94)	0.6,1.4

### Obstetric history and delivery outcome

The mean age of mother´s first marriage was 15.4 ± 3.2 years, and the mean age at first pregnancy were 18.6±2.8 years. Whereas, 851(36.5%) were pregnant before 18 years old. The participants had a mean pregnancy of 3.7±2.0 and the majority, 1232(52.8%) had noe-three pregnancies. During the last pregnancy, 1660(71.1%) mothers had at least one ANC visit. More than half, 1267 (54.3%) deliveries were at home and the majority of them, 874(37.5%) were assisted by traditional birth attendants. Only 249(10.7%) had at least on postnatal services (Table 2).

**Table 2:** obstetric history and delivery outcome of mothers during the last pregnancy, April 2019, northwest Ethiopia (p-value = 0.05)

Variables	Number (%)	95% CI
**Mother´s age at first pregnancy**		
12-17 years	851 (36.5)	34.5, 38.4
18-25 years	1432 (61.4)	59.3, 63.3
≥ 26 years	52 (2.3)	1.7, 2.9
**Number of pregnancies**		
1-3 Pregnancies	1232 (52.8)	50.7,54.8
4-6 Pregnancies	888 (38.0)	36.1,40.0
7-12 Pregnancies	215 (9.2)	8.1,10.5
**Number of living children**		
1-3 Children	1065 (52.8)	50.6, 55.0
4-6 Children	805 (39.9)	37.8, 42.1
7-11 Children	146 (7.2)	6.2, 8.5
**ANC visits**		
Yes	1660 (71.1	69.2, 72.9
No	675 (28.9	27.1, 30.8
**Gestational age**		
Preterm	19 (0.8)	0.52, 1.3
Term	2283 (98.4)	97.8, 98.8
Post term	18 (0.8)	0.5, 1.2
**Place of delivery**		
Home	1267 (54.3)	52.3, 56.3
Health facility	1010 (43.3)	41.3, 45.3
Other	57 (2.4)	1.9, 3.2
**Delivery assistant**		
Traditional birth attendants	874 (37.5)	35.5, 39.4
Mother in law/relatives	407 (17.4)	15.9, 19.0
Health extension workers	11 (0.5)	0.3, 0.8
Health center staff	762 (32.7)	30.8, 34.5
Hospital staff	222 (9.5)	8.4, 10.8
Others	58 (2.5)	1.9, 3.2
**Postnatal care**		
Yes	249 (10.7	9.5, 11.9
No	2085 (89.3)	88.0, 90.
**Newborn immunization status**		
No	1008 (43.2)	39.4-43.3
Yes	1326 (56.8)	54.8-58.8

### Level of mother´s knowledge on neonatal danger signs

Of the mothers, 1120 (48.0) (95%CI: 45.9, 50.0) have heard about neonatal danger signs. The source of information about neonatal danger signs were through health extension workers, 610 (26.1%) (95%CI: 24.4, 28.0), through friends, 498 (21.3%) (95%CI: 19.7, 23.0) and other healthcare workers such as medical doctors, nurses and laboratory technicians, 316 (13.5%) (95%CI: 12.2, 15.0). The overall knowledge of mothers on neonatal danger signs was 1509 (64.6) (95%CI: 62.7, 66.5). The details of mothers´ knowledge of neonatal danger signs are shown in Table 3.

**Table 3:** level of knowledge of mothers on neonatal danger signs, April 2019, northwest Ethiopia (p-value = 0.05)

Variable	Number (%)	95% CI (%)
**Have you heard about neonatal danger signs**		
Yes	1120 (48.0)	45.9, 50.0
No	1215 (52.1)	49.6, 53.7
**Source of information about neonatal danger signs**		
Health extension workers	610 (26.1)	24.4, 28.0
Other health professionals	316 (13.5)	12.2, 15.0
Family Health Card	163 (7.0)	6.0, 8.1
Radio	71 (3.0)	2.4, 3.8
Television	41 (1.8)	1.3, 2.4
Friends	498 (21.3)	19.7, 23.0
Others	37 (1.6)	1.2, 2.2
**Attendance on health education by HEW**		
Yes	825 (35.3)	33.4, 37.3
No	1510 (64.7)	62.5, 67.0
**Mothers´ knowledge on neonatal danger signs**		
Does not know any neonatal danger signs	826 (35.4)	33.5, 37.3
At least one danger sign	1243 (53.2)	51.2, 55.3
At least two danger signs	249 (10.7)	9.5, 12.0
At least three danger signs	15 (0.6)	0.4, 1.1
At least four danger signs	2 (0.1)	0.02, 0.3
**Overall knowledge of mothers on neonatal danger signs**		
Poor knowledge	826 (35.4)	33.5, 37.3
Good knowledge	1509 (64.6)	62.7, 66.5

About 160 (11.0%) mothers have experienced at least one of the danger signs on their baby. The frequently reported danger signs on a baby were hot feeling 106 (66.3%) (95% CI: 58.5, 73.2), fast breathing, 67(41.9) (95% CI: 34.4, 49.7), difficulty of breathing, 61(38.1%) (95%CI: 30.9, 46.0), and inability to suck or feed 41 (25.6) (95%CI: 19.4, 33.0). The details of mothers´ experience of neonatal danger signs are shown in Table 4.

**Table 4:** mother’s experience of neonatal danger signs, April 2019, northwest Ethiopia (N=1,472) (p-value = 0.05)

Variable	Number (%)	95%CI
**Mothers experience on neonatal danger signs**		
No experience	1312 (89.1)	87.4, 90.6
One	65 (4.4)	3.5, 5.6
Two	46 (3.1)	2.4, 4.2
Three	23 (1.6)	1.04, 2.3
Four	16 (1.1)	0.7, 1.8
Five	01 (0.1)	0.01, 0.5
Six	06 (0.4)	0.2, 0.9
Eight	01 (0.1)	0.01, 0.5
Nine	02 (0.1)	0.03, 0.5
**Mothers experience on neonatal danger signs**		
No experience	1312 (89.1)	87.4, 90.6
At least one experience	160 (10.9)	9.4,12.6
**Experienced danger signs (n=160)**		
Newborn feels hot	106 (66.3)	58.5, 73.2
Newborn feels cold	14 (8.8)	5.2, 14.3
Fast breathing	67(41.9)	34.4, 49.7
Difficulty of breathing	61(38.1)	30.9, 46.0
Swollen, red eyelids and pus discharging eyes	16(10)	6.2, 15.8
Redness, swollen and foul-smelling cord	23 (14.4)	9.7, 20.8
Abnormal or unusual body movement	6(3.8)	1.7, 8.2
Floppy or absence of movement unless stimulated	8 (5.0)	2.5, 9.8
Yellow soles and feet	4 (2.5)	0.9, 6.5
Inability to suck or feeds	41 (25.6)	19.4,33.0
Was the child preterm	11 (6.9)	3.8,12.1

### Age of danger sign noticed and treatment-seeking of mothers for their babies

Around 54 (49.1%) (95% CI: 39.7, 58.5) mothers have noticed neonatal danger signs of their babies within 24hours of delivery and 37 (33.6%) (95% CI: 25.3, 43.1) have noticed after 48 hours of delivery. Mothers noticed their baby´s danger signs at home after birth were 50 (45.5%) (95% CI: 36.3, 55.0), and 48 (43.6) 95% CI: 34.6, 53.2) have noticed at home immediately after birth. In this study, health centers were the first place for first treatment;72 (65.5%) (95% CI: 56.0, 73.9) of mothers for neonatal danger signs, followed by health posts, 17 (15.5%) (95% CI: 9.8, 23.6). Out of 110 mothers who reported the outcome of their neonates' experience with danger signs, 106 (96.4%) had improved health and three (2.7%) (95% CI: 0.9, 8.3) reported that their health deteriorated (Table 5).

**Table 5:** danger sign notice and treatment-seeking of mothers for their babies, April 2019, northwest Ethiopia (p-value = 0.05)

Variable	Number (%)	95%CI
**Time noticed danger signs (n=110)**		
≤ 24hours	54 (49.1)	39.7, 58.5
25-48hours	19 (17.3)	11.2, 25.7
≥ 48hours	37 (33.6)	25.3, 43.1
**Place noticed danger signs (n=110)**		
Health facility, immediately after birth	12 (10.9)	6.2, 18.4
Home, immediately after birth	48 (43.6)	34.6, 53.2
Home, after birth	50 (45.5)	36.3, 55.0
**Place of first treatment for neonatal danger signs (n = 110)**		
Health post	17 (15.5)	9.8, 23.6
Health center	72 (65.5)	56.0, 73.9
Hospitals	16 (14.6)	9.0, 22.6
Private clinic	4 (3.6)	1.4, 9.4
Home/others	1 (0.9)	0.1, 6.4
**Health professionals treated neonatal danger signs (n = 110)**		
Health extension workers	18 (16.4)	10.48, 24.6
Nurses	52 (47.3)	38.0, 56.7
Health officers	9 (8.2)	4.3, 15.1
Medical doctors	13 (11.8)	6.9, 19.4
Unknown	18 (16.4)	10.5, 24.6
**The outcome of neonatal danger signs (n = 110)**		
Health improved	106 (96.4)	90.6, 98.7
Health deteriorated	3 (2.7)	0.9, 8.3
Status unknown	1 (0.9)	0.1, 6.4

### Predictors of mother´s knowledge on neonatal danger signs

In this study, being semi-urban resident, AOR=1.87 (95% CI: 1.19, 2.94), mothers having an educational status of college and above, AOR=3.31 (95% CI: 1.06,10.27) and distances from the residence to the nearest health center within, 5.1-10 kilometer, AOR=1.79 (95% CI: 1.10, 2.91), and within, 15.1-28 kilometer, AOR=1.91 (95% CI: 1.12, 3.26) were the predictors of women knowledge on neonatal danger signs. The other predictors include newborn immunization status, AOR=1.30 (95% CI: 1.08, 1.58), and mothers experience on neonatal danger signs, poor experiences, AOR=0.65 (95% CI: 0.46,0.94) (Table 6).

**Table 6:** predictors of mothers´ knowledge on neonatal danger signs, April 2019, northwest Ethiopia (p-value = 0.05)

Variable	Knowledge on neonatal danger signs		COR (95% CI)	AOR (95% CI)
	Good	Poor		
**Residence**				
Urban	218	95	**1.32 (1.02,1.72)**	1.47 (0.89,2.43)
Semi-urban	221	114	1.12 (0.87,1.43)	1.87 (1.19,2.94)*
Rural	1070	617	**1**	**1**
**Mother´s education**				
Primary education	372	204	**0.41 (0.15,1.11)**	**1**
Secondary education	157	75	**0.48 (0.17,1.31)**	1.22 (0.9,1.7)*
College and above	22	5	**1**	3.31 (1.1,10.3)*
**Home distance to the nearest health center**				
≤ 1 kilometer	365	206	**1**	**1**
1.1-5 kilometer	439	294	**0.84 (0.67,1.06)**	0.96 (0.6,1.4)
5.1-10 kilometer	400	178	**1.26 (0.99,1.62)**	1.79 (1.1,2.9)*
15.1-28 kilometer	305	148	**1.16 (0.89,1.51)**	1.91 (1.12,3.26)*
**ANC attendance at Health Post**				
Yes	202	78	**1**	**1**
No	1307	748	**0.67 (0.51,0.89)**	0.70 (0.49,1.01)*
**Place of delivery**				
Home	813	454	**1**	**1**
Health facility	653	357	1.02 (0.86,1.21)	0.99 (0.81,1.22)
Other	43	14	**1.72 (0.93,3.17)**	1.82 (0.94,3.52)*
**Postnatal Care**				
Yes	146	103	**1**	**1**
No	1363	722	**1.33 (1.02,1.74)**	1.35 (0.99,1.83)*
**Newborn Immunization Status**				
Yes	831	495	**1**	**1**
No	678	330	**1.22 (1.03,1.45)**	1.30 (1.08,1.58)*
**Mothers experience on neonatal danger signs**				
Poor experience	1392	783	**0.65 (0.46,0.94)**	0.65 (0.46,0.94)*
Good experience	117	43	**1**	**1**

Note: * = p ≤ 0.05, ** = p ≤ 0.001

### Predictors of mother´s experience on neonatal danger signs

This study also revealed that being urban resident, AOR=1.77 (95% CI: 1.04, 3.02), mothers having seven-11 children, AOR=9.76 (95% CI: 2.26, 42.03), mothers having no obstetric danger sign or complication, AOR=0.36 (95% CI: 0.24, 0.55), and post-term gestational age, AOR=6.46 (95% CI: 2.13, 19.53) were the predictors of mothers experience on neonatal danger signs. The other predictors include preterm gestational age, AOR=3.34 (95% CI: 0.83, 13.43), assisted by hospital healthcare workers during delivery, AOR=2.10 (95% CI: 1.01, 4.32), and poor overall mother´s knowledge on neonatal danger signs, AOR=0.66 (95% CI: 0.46, 0.94) (Table 7).

**Table 7:** predictors of mother’s experience on neonatal danger signs, April 2019, northwest Ethiopia

Variable	Mothers experience		COR (95% CI)	AOR (95% CI)
	Good	Poor		
**Residence**				
Urban	34	279	2.04 (1.4,3.1)	1.77 (1.0,3.0)*
Semi-urban	31	304	1.71 (1.1,2.6)	1.54 (1.0,2.5)*
Rural	95	1592	1	**1**
**Father´s occupation**				
Farmer	130	1953	1	**1**
Merchant	10	87	1.72 (0.9,3.4)	1.31 (0.6,2.8)
Gov't employee	7	57	1.84 (0.8,4.1)	1.61 (0.7,3.8)
Others	13	78	2.50 (1.4,4.6)	2.28 (1.1,4.6*
**Number of pregnancies**				
1-2 Pregnancies	60	761	1.28 (0.9,1.9)	7.68 (1.7,34.2)**
3-4 Pregnancies	56	701	1.30 (0.9,2.0)	3.24 (1.31,8.1)*
5-12 Pregnancies	44	713	1	**1**
**Number of living children**				
1-3 Children	26	656	1	**1**
4-6 Children	58	685	2.14 (1.3,3.4)	4.5(1.4,14.5)*
7-11 Children	39	552	1.78 (1.7,3.0)	9.8(2.3,42.0)**
**ANC attendance**				
Yes	132	1528	1	
No	28	647	0.50 (0.3,0.8)	
**Gestational age**				
Preterm	4	15	3.77 (1.23,11.5)	3.34 (0.8,13.4)*
Term	151	2132	1	**1**
Post term	5	13	5.43 (1.91,15.4)	6.46 (2.1,19.5)**
**Delivery assistant**				
Traditional birth attendants	46	828	1	**1**
Mother in law/relatives	32	375	1.54 (1.0,2.5)	1.89 (1.1,3.2)*
Health center staff	58	704	1.48 (1.0,2.2)	0.91 (0.6,1.5)
Hospital staff	17	205	1.49 (0.8,2.7)	2.10 (1.0,4.3)*
Others (HEW, etc.)	7	62	2.03 (0.9,4.7)	2.62 (1.1,6.3)*
**Overall knowledge of mothers on neonatal danger signs**				
Poor knowledge	43	783	0.65 (0.46,0.94)	0.66 (0.46,0.94)*
Good knowledge	117	1392	1	**1**

Note: * = p ≤ 0.05, ** = p ≤ 0.001

## Discussion

In this study, the overall knowledge of mothers´ on neonatal danger signs was high. About 160 (11.0%) mothers have experienced at least one of the danger signs appeared on their baby. Fifty percent of mothers have noticed neonatal danger signs on their babies within ≤ 24 hours of age. Most of the mothers have noticed their babies´ danger signs at home after birth. The frequently reported danger signs noticed on the baby were baby feels hot, fast breathing, and difficulty of breathing. Being semi-urban resident, mother's educational status of college and above, distances from home to the nearest health center within, 5.1-10 kilometer, and within, 15.1-28 kilometer, newborn immunization status, and mother's experience on neonatal danger signs were found to predict mothers knowledge on neonatal danger signs. This study revealed that most of the mothers, (64.6%) mentioned at least one or more neonatal danger signs. This finding was inline with the research done in Ambo (70.0%) and Mekelle (50.6%) in Ethiopia, and in Uganda (58.3) [9, 10, 23]. However, it is lower than a study done in Northwest Ethiopia (79.8%) and higher than the finding in the Kenyan study (15.5%) [11, 12]. The main reasons for these differences include the operational definition of good knowledge on neonatal danger signs, method of data collection and differences in healthcare during delivery. In some studies mentioning one neonatal danger sign was considered as good knowledge while others used more than two. Moreover, unlike our study, some researchers used ‘yes/no´ questions for data collection which is easier to answer than listing. On the other hand, only 11.4% of mothers in our study listed more than one neonatal danger sign, which is lower than most studies mentioned above. This finding may also suggest why neonatal mortality is still high in Ethiopia. It was found that the main sources of information were through health extension workers. Researchers in Ambo and northwest Ethiopia have observed similar findings [10, 11]. Since our study is a community based, where most (72.3%) of the participants were from rural settings, the primary healthcare delivery depends on community health workers. Interventions to improve the mothers' knowledge of neonatal danger signs should also consider the capacity building of community health workers.

The study further showed that only a few (4.4%) mothers have experienced on neonatal danger signs. This finding was collerated with the study conducted in Ambo, Ethiopia (5.4%). Fever was the most commonly experienced danger signs in our study and in most of the other studies reviewed except a study in Uganda in which fast breathing is more frequently mentioned [10, 12, 18, 25, 26]. This is because fever is easier to identify than other neonatal danger signs. The difference might be due to the prevalence of acute febrile illnesses between the study areas. Most of the mothers in our study first sought medical care from health centers unlike research done in urban India where infants were taken to Medical doctors [18]. The difference in healthcare system delivery and research settings are possible reasons for this difference. For effective reduction in neonatal mortality, health centers need to have neonatal units with well-trained healthcare workers to treat or timely refer neonates with neonatal danger signs. We have found that predictor for good knowledge on neonatal danger signs included; living in semi-urban or near to health center, maternal education, and previous experience of neonatal danger signs. Reasonably, maternal education was consistently associated with good knowledge of neonatal danger signs in most research findings [5, 10, 13, 21]. Empowering and educating women should take the paramount strategy to reduce neonatal mortality. Other associated factors in our literature search included postnatal care, having read MCH booklet, and previous experience of danger signs [10, 12]. Hence, the inclusion of neonatal danger signs in the MCH booklet can increase awareness.

ANC was not significantly associated with increased knowledge in our study. There are mixed findings in other studies. For example, in a study conducted in Ethiopia, ANC was a significant factor unlike a study done in Uganda [9, 11]. These differences may be due to the inclusion of neonatal danger signs in health education during ANC follow up. Since most mothers have attended ANC at least once, including neonatal health might leverage the knowledge of mothers. The high knowledge of mothers on neonatal danger signs was very interested in the study area, as knowing those general danger signs are important for early detection of serious illness and seeking healthcare for their child. This has to be strengthened. However, the real experience of mothers on neonatal danger signs was low. Provision of health education on neonatal danger signs and newborn immunization to rural residents by considering their home distance and experience to improve their knowledge and healthcare-seeking behavior were the key areas of intervention. Health promotion and education on the experience on neonatal danger signs by considering residence, family size, obstetric danger signs or complication, gestational age, delivery assistant, newborn weight, and mother´s knowledge on neonatal danger signs are very important. Study limitations: We have included only mothers who delivered in the past six months, which can reduce recall bias. We have used a cross-sectional design that did not help for casual relationship establishment. As most of our participants (64.2%) were never attended education, there is a possibility that some women may get difficult to understand our questionnaire.

## Conclusion

The knowledge of mothers on neonatal danger signs was high. Residence, mother's educational status, distances from the nearest health center, immunization status, and mothers experience on neonatal danger signs were significantly associated with mothers´ knowledge of neonatal danger signs. On the other hand, this study found that the real experience of mothers on neonatal danger signs was low. Residence, family size, obstetric danger signs or complication, gestational age, assisted delivery, newborn weight, and knowledge on neonatal danger signs were predictors of mother's experience on neonatal danger signs. Since our quantitative cross-sectional study did not allow us to analyze the proximal and distal causes of this “know-do” gap, we recommend further implementation and qualitative study in this area.

### What is known about this topic

- There is significant decline in neonatal mortality globally, however it remained unacceptably high in Ethiopia;
- Evidence showed that most mothers know at least one neonatal danger sign.

### What this study adds

- The conversion rate of mothers´ knowledge to practice is still low;
- Living in semi-urban area, living within 5.1-10 kilometer from the nearest health center, mothers having an educational status of college and above, having immunized newborn, and having experience on neonatal danger signs were the predictors of women knowledge;
- Being an urban resident, having 7-Eleven children, having no obstetric danger sign, assisted by hospital healthcare worker, preterm gestational age, post-term gestational age, and poor overall mother´s knowledge were the predictors of mother's experience on neonatal danger signs.
